# Comparison of the effects of fibrates versus statins on plasma lipoprotein(a) concentrations: a systematic review and meta-analysis of head-to-head randomized controlled trials

**DOI:** 10.1186/s12916-017-0787-7

**Published:** 2017-02-03

**Authors:** Amirhossein Sahebkar, Luis E. Simental-Mendía, Gerald F. Watts, Maria-Corina Serban, Maciej Banach, Lipid and Blood Pressure Meta-analysis Collaboration (LBPMC) Group

**Affiliations:** 1 Biotechnology Research Center, Mashhad University of Medical Sciences, Mashhad, Iran; 2 School of Medicine, University of Western Australia, Perth, Australia; 3 Biomedical Research Unit, Mexican Social Security Institute, Durango, Mexico; 4 Lipid Disorders Clinic, Department of Cardiology, Royal Perth Hospital, Perth, Australia; 5 Department of Functional Sciences, Discipline of Pathophysiology, “Victor Babes” University of Medicine and Pharmacy, Timisoara, Romania; 6 Department of Hypertension, Chair of Nephrology and Hypertension, Medical University of Lodz, Lodz, Poland

**Keywords:** Coronary heart disease, PPAR-α, 3-hydroxy-3-methyl-glutaryl-CoA, Apolipoprotein(a), Randomized controlled trial, Combination therapy

## Abstract

**Background:**

Raised plasma lipoprotein(a) (Lp(a)) concentration is an independent and causal risk factor for atherosclerotic cardiovascular disease. Several types of pharmacological approaches are under evaluation for their potential to reduce plasma Lp(a) levels. There is suggestive evidence that statins and fibrates, two frequently employed lipid-lowering drugs, can lower plasma Lp(a). The present study aims to compare the efficacy of fibrates and statins in reducing plasma concentrations of Lp(a) using a meta-analysis of randomized head-to-head trials.

**Methods:**

Medline and Scopus databases were searched to identify randomized head-to-head comparative trials investigating the efficacy of fibrates versus statins in reducing plasma Lp(a) levels. Meta-analysis was performed using a random-effects model, with inverse variance weighted mean differences (WMDs) and 95% confidence intervals (CIs) as summary statistics. The impact of putative confounders on the estimated effect size was explored using random effects meta-regression.

**Results:**

Sixteen head-to-head comparative trials with a total of 1388 subjects met the eligibility criteria and were selected for this meta-analysis. Meta-analysis revealed a significantly greater effect of fibrates versus statins in reducing plasma Lp(a) concentrations (WMD, –2.70 mg/dL; 95% CI, –4.56 to –0.84; *P* = 0.004). Combination therapy with fibrates and statins had a significantly greater effect compared with statin monotherapy (WMD, –1.60 mg/dL; 95% CI, –2.93 to –0.26; *P* = 0.019) but not fibrate monotherapy (WMD, –1.76 mg/dL; 95% CI, –5.44 to +1.92; *P* = 0.349) in reducing plasma Lp(a) concentrations. The impact of fibrates versus statins in reducing plasma Lp(a) concentrations was not found to be significantly associated with treatment duration (*P* = 0.788).

**Conclusions:**

Fibrates have a significantly greater effect in reducing plasma Lp(a) concentrations than statins. Addition of fibrates to statins can enhance the Lp(a)-lowering effect of statins.

## Background

Aside from low-density lipoprotein cholesterol (LDL-C) and triglycerides, lipoprotein(a) (Lp(a)) is an important contributor to atherogenesis [1]. Lp(a) is a lipoprotein particle comprised of an LDL domain and a covalently bound apolipoprotein(a) (apo(a)). Lp(a) is characterized by a protein content of 26–31%, a long half-life, and an atherothrombotic effect likely due to its selective accumulation within atherosclerotic plaque and its inhibition of the fibrinolytic pathway [2]. Plasma concentrations of Lp(a) are an independent risk factor for early atherosclerotic cardiovascular disease [3–5]. Lp(a) is a low-density lipoprotein-like substance with a core of cholesteryl esters and a surface layer of phospholipids and unesterified cholesterol that contains a single molecule of apolipoprotein B-100 bound to a molecule of apo(a) by a disulfide linkage [6]. Values of plasma Lp(a) levels of more than 30 mg/dL are considered elevated [7, 8], and are associated with increased risk of atherogenesis and cardiovascular disease, especially when exceeding 50 mg/dL [9]. A single molecule of apo(a) is secreted by the liver and has a structure similar to plasminogen but without protease activity [10]. In addition to the atherogenic properties afforded by the presence of apoB-100, the apo(a) component of Lp(a) confers thrombogenic effects to the particle [11]. A growing body of evidence for an atherogenic and pro-thrombotic effect of Lp(a) has been reported, as well as its likely causal association with risk of coronary heart disease and stroke [12, 13]. Thus, therapeutic strategies to reduce plasma Lp(a) concentrations in patients with hyper-Lp(a) are particularly important to reduce cardiovascular mortality. In this regard, various therapeutic interventions for lowering Lp(a) levels have been reported, including apheresis techniques, nicotinic acid, statins, fibrates, and aspirin, among others [14–17]. It has been reported that plasma Lp(a) levels are decreased by monoclonal antibodies targeting proprotein convertase subtilisin/kexin type 9 (PCSK9) [18–21]. PCSK9 inhibitors act by increasing the density of LDL receptors on the surface of hepatocytes, which subsequently causes a marked reduction of plasma LDL and LDL-apoB [22–24]. Since the availability of LDL-apoB plays a key role in the formation of Lp(a) particles [25], LDL-lowering activity of PCSK9 inhibitors is accompanied by a significant fall in plasma Lp(a) levels, as suggested by pooled analyses and meta-analyses [22–24]. Several lines of clinical evidence have also shown that statins and fibrates, as the most widely used lipid-lowering drug classes, can lower plasma Lp(a) concentrations [14]. However, evidence from comparative trials has not been conclusive.

Mixed dyslipidemia is characterized by high serum concentrations of total and LDL-C as well as of triglycerides [26]. Statins and fibrates are among the first-line pharmacotherapies for mixed dyslipidemia. Findings of clinical trials have shown that the combination of statins and fibrates results in a significantly greater reduction in LDL-C and triglyceride levels and greater increases in high-density lipoprotein cholesterol (HDL-C) compared with monotherapy with either drug [27]. In addition, both statins and fibrates have been shown to reduce cardiovascular morbidity and mortality [28, 29]. Moreover, these classes of drug affect different aspects of lipoprotein metabolism. Fibrates decrease serum levels of cholesterol and triglycerides and increase HDL-C levels in hyperlipidemic patients, thereby reducing the risk of developing atherosclerosis [30]. The main mechanisms of action of fibrates are induction of lipoprotein lipolysis [31], induction of hepatic fatty acid uptake and reduction of hepatic triglyceride production [32, 33], enhancement of hepatic removal of LDL particles [34], reduction of plasma triglyceride-rich lipoproteins [35], and elevation of HDL production [36].

Statins mainly act through enhancement of plasma clearance of LDL and reduction of hepatic very low-density lipoprotein production [37]. Statins reduce hepatic cholesterol biosynthesis through inhibition of 3-hydroxy-3-methyl-glutaryl-CoA reductase, causing depletion of intracellular cholesterol content and resulting in an increase in the expression and density of hepatic LDL receptors [38].

Owing to the importance of Lp(a) as an emerging coronary risk factor, and the wide use of statins and fibrates in the management dyslipidemias, the present study aimed to compare the effects of these two classes of drugs on plasma Lp(a) concentrations through a systematic review and meta-analysis of head-to-head clinical trials. A secondary aim was to assess if combination therapy with statins and fibrates is associated with a greater effect on plasma Lp(a) levels compared with monotherapy with either of the agents.

## Methods

### Search strategy

This study was designed in accordance with the instructions of the 2009 preferred reporting items for systematic reviews and meta-analysis (PRISMA) statement [39]. SCOPUS (http://www.scopus.com) and Medline (http://www.ncbi.nlm.nih.gov/pubmed) databases were searched using the following search terms in titles and abstracts (also in combination with MESH terms): (rosuvastatin OR pravastatin OR fluvastatin OR simvastatin OR atorvastatin OR pitavastatin OR lovastatin OR cerivastatin) AND (fenofibrate OR bezafibrate OR clofibrate OR ciprofibrate OR gemfibrozil OR “fibric acid” OR “clofibric acid” OR procetofen) AND (lipoprotein(a) OR “lipoprotein (a)” OR Lp(a) OR “Lp (a)”). The wild-card term “*” was used to increase the sensitivity of the search strategy. The search was limited to studies in human. The literature was searched from inception to October 3, 2016.

### Study selection

Trials comparing the effects of statins versus fibrates on serum/plasma concentrations of Lp(a) were included in this meta-analysis. Non-interventional studies and studies not providing sufficient information on baseline or follow-up Lp(a) concentrations were excluded from the meta-analysis. Before excluding a study for the latter, the author(s) were contacted and asked to provide the necessary data.

### Quality assessment

Risk of bias in the studies considered in this meta-analysis was evaluated according to the Cochrane instructions [40]. Selection bias, performance bias, attrition bias, detection bias, reporting bias, and other sources of bias were judged to be high, low, or unclear in each of the included studies.

### Data extraction

Studies meeting the inclusion criteria were reviewed and data regarding authors, study location, publication date, number of studied population, trial design, dose and duration of intervention, control group allocation, baseline characteristics of studied population (including age, sex, systolic and diastolic blood pressure, body mass index (BMI), and plasma lipid concentrations), and changes in plasma concentrations of Lp(a). When the values were only presented graphically, GetData Graph Digitizer 2.24 software (http://getdata-graph-digitizer.com/) was used to digitize and extract the data.

### Quantitative data synthesis

Comprehensive Meta-Analysis (CMA) V2 software (Biostat, NJ) [41] and Review Manager, version 5.2 (Cochrane Collaboration) were used for statistical procedures. All reported Lp(a) concentrations were harmonized in mg/dL. Inverse variance-weighted standardized mean difference and 95% confidence intervals (CIs) were used as the summary statistic, considering a correlation coefficient (R) of 0.5. Conversion of median and inter-quartile range to mean and standard deviation was performed as suggested by Hozo et al. [42]. When plasma Lp(a) levels were presented in multiple time points, data belonging to the longest duration of treatment was included in the meta-analysis. Meta-analysis was performed using a random-effects model (using DerSimonian–Laird method) and the generic inverse variance weighting method. Heterogeneity was quantitatively assessed using *I* ^*2*^ index and Cochrane Q. Sensitivity analysis was performed using the leave-one-out method [43–46]. A subgroup analysis was conducted to explore the impact of treatment duration (<12 weeks vs. ≥ 12 weeks) on plasma Lp(a) concentrations.

### Meta-regression

Random effects meta-regression was performed using the unrestricted maximum likelihood method to evaluate the association between calculated weighted mean differences (WMD) in plasma Lp(a) concentrations and duration of treatment.

### Publication bias

Presence of publication bias in the meta-analysis was investigated using assessment of Begg’s funnel plot and statistical tests as previously described [47, 48]. The “trim and fill” method was used to adjust the effect size for potential publication bias [49].

## Results

### Flow of included studies

**Fig. 1:**
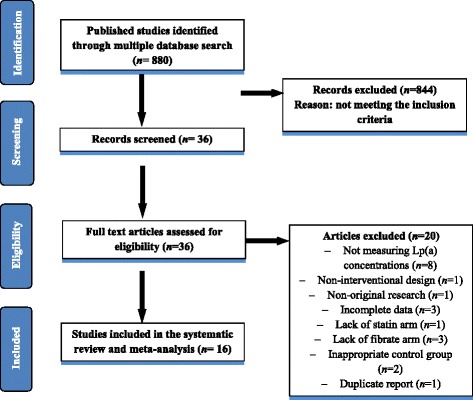
Flow chart of the number of studies identified and included into the meta-analysis

Briefly, after multiple database searches, 880 published studies were identified and the abstracts reviewed; 844 did not meet the inclusion criteria and were excluded. Next, 36 full text articles were carefully assessed and reviewed, of which 20 studies were excluded for not measuring Lp(a) concentrations (*n* = 8), having a non-interventional design (*n* = 1), being non-original research (*n* = 1), presenting incomplete data (*n* = 3), lack of statin treatment arm (*n* = 1), lack of fibrate treatment arm (*n* = 3), having an inappropriate control group (*n* = 2), and duplicate reporting (*n* = 1). Finally, 16 studies with 19 treatment arms were found to be eligible and included in the systematic review and meta-analysis. The study selection process is shown in Fig. 1.

### Characteristics of included studies

A total of 1388 individuals were recruited in the 15 randomized controlled studies, including 588, 587, and 213 subjects in the fibrate monotherapy, statin monotherapy, and statin/fibrate combination therapy arms (participants of the cross-over trials were considered in both fibrate and statin monotherapy arms), respectively (Table 1). Included studies were published between 1994 and 2009. The clinical trials used different types and doses of fibrates and statins and evaluated atorvastatin 10 mg/day (*n* = 2) [50, 51], atorvastatin 20 mg/day (*n* = 1) [52], atorvastatin 40 mg/day (*n* = 1) [53], simvastatin 10 mg/day (*n* = 1) [54], simvastatin 20 mg/day (*n* = 5) [55–59], pravastatin 40 mg/day (*n* = 1) [60], lovastatin 10 mg/day (*n* = 1) [61], lovastatin 40–80 mg/day (n = 1) [62, 63], fluvastatin 40 mg/day (*n* = 1) [64], rosuvastatin 10 mg/day (*n* = 1) [65], fenofibrate 200 mg/day (*n* = 5) [50–52, 59, 65], fenofibrate 145 mg/day (*n* = 1) [53], fenofibrate 160 mg/day (*n* = 1) [57], gemfibrozil 1200 mg/day (*n* = 6) [54, 55, 58, 60, 62, 63], and bezafibrate 400 mg/day (*n* = 3) [56, 61, 64]. The range of intervention periods was from 8 weeks [60, 61] to 24 weeks [52]. Study designs of included studies were cross-over [51, 54, 60, 61] and parallel-group [53, 55–59, 62–65]. Selected trials enrolled subjects with diabetes [52, 54, 57], combined hyperlipidemia [50–59, 62, 63], familial defective apoB [60], heart transplantation [61], primary dyslipoproteinemia [54], mixed dyslipidemia combined with hyper-Lp(a) [62], primary hypercholesterolemia combined with hyper-Lp(a) [63], primary hypercholesterolemia [64, 65], and primary hypertriglyceridemia [65].

**Table 1:** Demographic characteristics of the included studies

Author	Study design	Target Population	Treatment duration	*n*	Study groups	Age, years	Female, *n* (%)	BMI, kg/m^2^	Total cholesterol, mg/dL	LDL-C, mg/dL	HDL-C, mg/dL	Triglycerides, mg/dL	Lp(a), mg/dL	Lp(a) change, mg/dL
Athyros et al. (2002) [52]	Randomized, open-label trial	Type 2 diabetes and combined hyperlipidemia	24 weeks	40 40 40	Atorvastatin 20 mg/day Fenofibrate 200 mg/day Atorvastatin 20 mg/day + fenofibrate 200 mg/day	57 (44–67)^a^ 58 (48–69)^a^ 58 (50–68)^a^	17 (42.5) 18 (45.0) 17 (42.5)	ND ND ND	252 ± 17 253 ± 17 255 ± 19	161 ± 15 163 ± 15 163 ± 16	34.6 ± 3.2 34.8 ± 3.4 35.1 ± 3.5	278 ± 24 281 ± 24 278 ± 23	18.4 ± 3.7 20.1 ± 5.2 19.2 ± 4.1	2.0 –4.0 1.0
Bredie et al. (1996) [55]	Randomized, double-blind, placebo-controlled trial	Familial combined hyperlipidemia	12 weeks	41 40	Simvastatin 20 mg/day Gemfibrozil 1200 mg/day	50.4 ± 10.8 53.4 ± 9.4	9 (21.9) 14 (35.0)	26.6 ± 2.7 27.2 ± 3.0	282.2 ± 37.1 290.0 ± 37.8	149.6 ± 37.8 157.7 ± 38.2	32.4 ± 7.3 35.1 ± 7.7	283.4 ± 106.2 285.2 ± 108.0	19.6^b^ 18.0^b^	6.5 2.7
Hansen et al. (1994) [60]	Randomized, cross-over trial	Familial defective apolipoprotein B-100	8 weeks	17 13	Pravastatin 40 mg/day Gemfibrozil 1200 mg/day	45.8 ± 17.1 40.8 ± 12.3	12 (70.5) 7 (53.8)	ND ND	320.9 ± 58.0 320.9 ± 46.4	247.4 ± 54.1 235.8 ± 42.5	46.4 ± 7.7 50.2 ± 19.3	115.1 (79.7–168.2)^a^ 141.7 (106.2–230.2)^a^	10.5 (3.5–16.7)^a^ 8.3 (3.4–12.9)^a^	0.9 1.1
Perez-Jimenez et al. (1995) [61]	Randomized, cross-over trial	Patients with heart transplant	8 weeks	18 18	Lovastatin 10 mg/day Bezafibrate 400 mg/day	54 ± 2	2 (11.1)	ND	302 ± 7 307 ± 5	213 ± 5 212 ± 5	54 ± 3 51 ± 4	170 ± 15 201 ± 17	34 ± 9 37 ± 9	5.0 –13.0
Melenovsky et al. (2002) [51]	Randomized, open-label, cross-over trial	Combined hyperlipidemia	10 weeks	15 14	Fenofibrate 200 mg/day Atorvastatin 10 mg/day	48.0 ± 6.9 46.4 ± 8.9	ND ND	27.7 ± 2.3 27.9 ± 3.2	300.0 ± 56.4 283.0 ± 35.9	174.7 ± 46.4 165.8 ± 23.2	49.4 ± 8.8 47.9 ± 15.8	492.4 ± 440.2 465.0 ± 372.8	24 ± 29 17 ± 23	2.8 1.8
Ohrvall et al. (1995) [54]	Randomized, double-blind, cross-over trial	Diabetes and hyperlipo-proteinemia	4 months	25 24	Gemfibrozil 1200 mg/day Simvastatin 10 mg/day	63.7 (48–78)^a^	9 (31.0)	ND	232.0 ± 40.2	152.7 ± 43.3	36.7 ± 6.9	116.0 ± 52.5	37.77 ± 54.75	–7.0 –2.4
Ramires et al. (1995) [62]	Randomized clinical trial	Hyperlipidemia and hyperlipo-proteinemia	12 weeks	14 13	Gemfibrozil 1200 mg/day Lovastatin 40-80 mg/day	54 ± 7 55 ± 9	5 (55.5) 3 (30.0)	ND ND	298.9 ± 12.3 299.3 ± 11.2	209.9 ± 20.8 208.0 ± 11.9	31.7 ± 7.3 34.4 ± 6.1	295.8 ± 23.0 281.6 ± 20.3	29.8 ± 5.7 27.8 ± 4.1	–7.5 1.4
Bairaktari et al. (1999) [50]	Open-label trial	Mixed hyperlipidemia	16 weeks	45 46	Atorvastatin 10 mg/day Fenofibrate 200 mg/day	49 ± 8 46 ± 10	16 (35.5) 15 (32.6)	26.8 ± 4 27.4 ± 3	278 ± 34 282 ± 37	198 ± 29 199 ± 36	40 ± 11 36 ± 16	271 ± 59 269 ± 60	14 ± 12 11.8 ± 10.5	1.2 –1.8
Davidson et al. (2009) [53]	Randomized, double-blind trial	Dyslipidemia	12 weeks	74 73 73	Atorvastatin 40 mg/day Fenofibrate 145 mg/day Atorvastatin 40 mg/day + fenofibrate 100 mg/day	56.3 ± 9.8 56.4 ± 10.5 54.9 ± 10.7	39 (52.7) 33 (45.2) 33 (45.2)	ND ND ND	254.4 ± 44.2 251.9 ± 47.5 252.0 ± 40.1	165.0 ± 37.7 166.6 ± 46.7 156.2 ± 33.6	42.7^b^ 41.2^b^ 43.3^b^	265.1^b^ 227.3^b^ 270.2^b^	69.0 ± 67.5 66.0 ± 65.3 76.8 ± 74.5	9.3 5.1 6.9
Greten et al. (1994) [64]	Randomized, double-blind trial	Primary hypercholesterolemia	12 weeks	64 67	Fluvastatin 40 mg/day Bezafibrate 400 mg/day	53.0 (18–75)^a^ 51.8 (22–70)^a^	37 (57.8) 37 (55.2)	24.6 (18.6–31.2)^a^ 24.7 (16.9–31.6)^a^	352.7 ± 89.4 340.3 ± 76.7	268.9 ± 88.5 257.4 ± 75.6	55.2 ± 12.2 55.2 ± 12.6	143.2 ± 48.0 138.0 ± 59.4	12.0 ± 16.5 14.0 ± 30.0	1.0 0.4
Kehely et al. (1995) [56]	Randomized, double-blind trial	Mixed hyperlipidemia	3 months	53 27 26	Placebo Simvastatin 20 mg/day Bezafibrate 400 mg/day	ND 52 ± 9 49 ± 10	ND 11 (40.7) 4 (15.3)	ND ND ND	298.5 ± 51.8	189.0 ± 54.9	42.5 ± 8.1	286.9 ± 122.2	19.8 ± 2.8	5.7 –2.5
May et al. (2008) [57]	Randomized, double-blind, placebo-controlled trial	Diabetes and mixed dyslipidemia	12 weeks	100 100 100	Fenofibrate 160 mg/day Simvastatin 20 mg/day Fenofibrate 160 mg/day + Simvastatin 20 mg/day	61.6 ± 11.5	135 (45.0)	ND	ND ND ND	ND ND ND	ND ND ND	ND ND ND	4.0 (2.0–6.0)^a^ 4.0 (2.0–6.0)^a^ 5.0 (3.0–9.0)^a^	1.0 1.0 0.0
Ramires et al. (1997) [63]	Randomized clinical trial	Hypercholesterolemia and hyperlipoproteinemia	12 weeks	14 13	Gemfibrozil 1200 mg/day Lovastatin 40-80 mg/day	54 ± 7 55 ± 9	5 (36.0) 6 (59.0)	ND ND	306 ± 13 307 ± 11	215 ± 21 213 ± 12	33 ± 7 35 ± 6	294 ± 23 280 ± 20	51 ± 10 48 ± 7	–13.0 –2.0
Saougos et al. (2007) [65]	Clinical trial	Hyperlipidemia	2 months	50 50	Rosuvastatin 10 mg/day Fenofibrate 200 mg/day	54.6 ± 14.6 55.9 ± 11	31 (62.0) 30 (60.0)	25.8 ± 4.2 34.3 ± 7	297.7 ± 50.2 235.8 ± 34.8	208.8 ± 42.5 146.9 ± 34.8	58.0 ± 11.6 50.2 ± 11.6	141.7 ± 53.1 239.1 ± 53.1	4.0 (2.0–7.4)^a^ 3.8 (2.0–7.9)^a^	0.0 0.1
Vigna et al. (1999) [58]	Randomized, double-blind trial	Men with mixed hyperlipidemia	2 months	15 15	Gemfibrozil 1200 mg/day Simvastatin 20 mg/day	53.6 ± 11.7 50.9 ± 9.9	0 (0.0) 0 (0.0)	26.7 ± 1.9 25.0 ± 2.5	280.2 ± 30.1 281.2 ± 38.8	208.5 ± 30.1 206.7 ± 37.8	46.1 ± 9.9 44.4 ± 8.2	46.1 ± 9.9 44.4 ± 8.2	25.7 ± 22.6 9.2 ± 11.8	–4.3 2.1
de Lorgeril et al. (1999) [59]	Randomized, double-blind trial	Dyslipidemic coronary patients	12 weeks	32 32	Fenofibrate 200 mg/day Simvastatin 20 mg/day	ND	ND	ND	278.4 ± 27.0 278.4 ± 27.0	193.3 ± 27.0 197.2 ± 23.2	46.4 ± 11.6 46.4 ± 7.7	186.0 ± 79.7 168.2 ± 88.5	34 ± 62 32 ± 39	–6.0 –2.0

Values are expressed as mean ± SD

^a^Median (interquartile range)

^b^Mean only

*BMI* body mass index, *ND* no data

### Lp(a) assay methods

Different assays methods were used to measure plasma Lp(a) concentrations. On this regard, some studies [50, 52, 56, 61, 65] measured Lp(a) levels in plasma using an enzyme-linked immunosorbent assay with a monoclonal anti-Lp(a) antibody (Terumo Medical, Elktron, MD). Other trials [54, 55, 60, 62, 63] determined Lp(a) concentrations by measuring the apoprotein(a) moiety in a commercially solid-phase two-site immunoradiometric assay using two different specific anti-apoprotein(a) monoclonal antibodies (Pharmacia, Uppsala, Sweden). Melenovky et al. [51] measured serum levels of Lp(a) by Laurell rocket immunoelectrophoresis using a commercial antisera (Immuno, Austria). Davidson et al. [53] measured Lp(a) concentrations by nuclear magnetic resonance (LipoScience Inc., Raleigh, North Carolina). Vigna et al. [58] determined Lp(a) levels by enzyme-linked immunosorbent assay with a polyclonal anti-apoprotein(a) antibody (Italiana Laboratori Bouty S.p.A., Milan, Italy). Three studies did not specify the method used to determine plasma Lp(a) concentrations [57, 59, 64]. Finally, all included studies were characterized by a lack of sufficient information regarding the allele-specific assay (Table 2).

**Table 2:** Methods used to measure Lp(a) in included studies

Study	Method	Kringle assay
Athyros et al. (2002) [52]	ELISA	NS
Bredie et al. (1996) [55]	IRA	NS
Hansen et al. (1994) [60]	IRA	NS
Perez-Jimenez et al. (1995) [61]	ELISA	NS
Melenovsky et al. (2002) [51]	IEP	NS
Ohrvall et al. (1995) [54]	IRA	NS
Ramires et al. (1995) [62]	IRA	NS
Bairaktari et al. (1999) [50]	ELISA	NS
Davidson et al. (2009) [53]	NMR	NS
Greten et al. (1994) [64]	NS	NS
Kehely et al. (1995) [56]	ELISA	NS
May et al. (2008) [57]	NS	NS
Ramires et al. (1997) [63]	IRA	NS
Saougos et al. (2007) [65]	ELISA	NS
Vigna et al. (1999) [58]	ELISA	NS
de Lorgeril et al. (1999) [59]	NS	NS

*ELISA* enzyme-linked immunosorbent assay, *IRA* immunoradiometric assay, *IEP* immunoelectrophoresis, *NMR* nuclear magnetic resonance, *NS* not specified

### Quality assessment

Most of the included studies were characterized by lack of information about the random sequence generation, allocation concealment, blinding of outcome assessment, and blinding of participants and personnel. On this regard, several trials showed high risk of bias for blinding of participants and personnel. Also, some studies had other biases related with the study design. However, almost all evaluated studies showed low risk of bias according to selective outcome reporting. Details of the quality of bias assessment are shown in Table 3.

**Table 3:** Quality of bias assessment of the included studies according to the Cochrane guidelines

Study	Random sequence generation	Allocation concealment	Selective reporting	Other bias	Blinding of participants and personnel	Blinding of outcome assessment	Incomplete outcome data
Athyros et al. (2002) [52]	L	U	L	L	H	U	L
Bredie et al. (1996) [55]	U	U	L	L	U	U	L
Hansen et al. (1994) [60]	U	U	L	L	H	U	L
Perez-Jimenez et al. (1995) [61]	U	U	L	U	H	U	L
Melenovsky et al. (2002) [51]	H	U	L	U	H	U	L
Ohrvall et al. (1995) [54]	U	U	L	L	U	U	U
Ramires et al. (1995) [62]	U	U	L	H	H	U	U
Bairaktari et al. (1999) [50]	H	U	L	U	U	U	L
Davidson et al. (2009) [53]	L	L	L	L	L	L	L
Greten et al. (1994) [64]	U	U	L	U	U	U	L
Kehely et al. (1995) [56]	U	U	L	L	U	U	L
May et al. (2008) [57]	L	U	L	U	L	U	U
Ramires et al. (1997) [63]	U	U	L	U	U	U	L
Saougos et al. (2007) [65]	H	U	L	U	U	U	L
Vigna et al. (1999) [58]	U	U	L	U	U	U	L
de Lorgeril et al. (1999) [59]	U	U	U	U	U	U	U

*L* low risk of bias, *H* high risk of bias, *U* unclear risk of bias

### Quantitative data synthesis

#### Fibrate monotherapy versus statin monotherapy

**Fig. 2:**
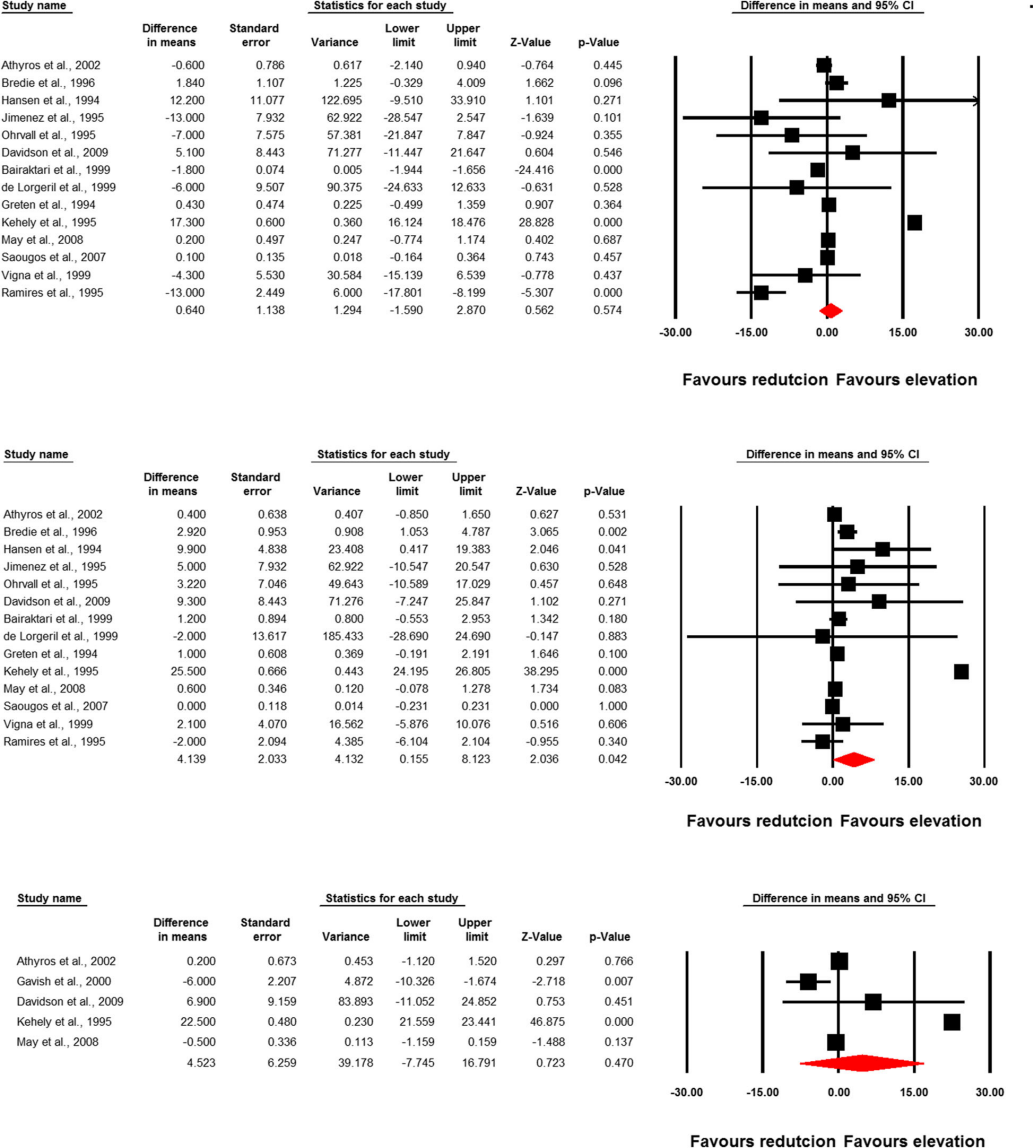
Forest plot detailing weighted mean difference and 95% confidence intervals for the impact of fibrates (upper plot), statins (middle plot), and statin/fibrate combinations (lower plot) on plasma Lp(a) concentrations in single-arm uncontrolled trials

**Fig. 3:**
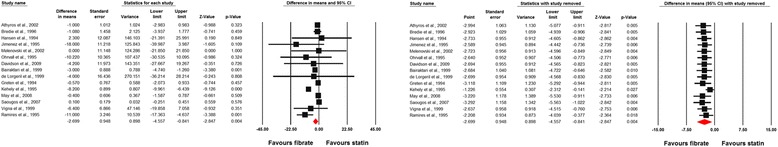
Forest plot detailing weighted mean difference and 95% confidence intervals for the impact of fibrate versus statin monotherapy on plasma Lp(a) concentrations. Right plot shows leave-one-out sensitivity analysis of the impact of fibrate versus statin monotherapy on plasma Lp(a) concentrations

**Fig. 4:**
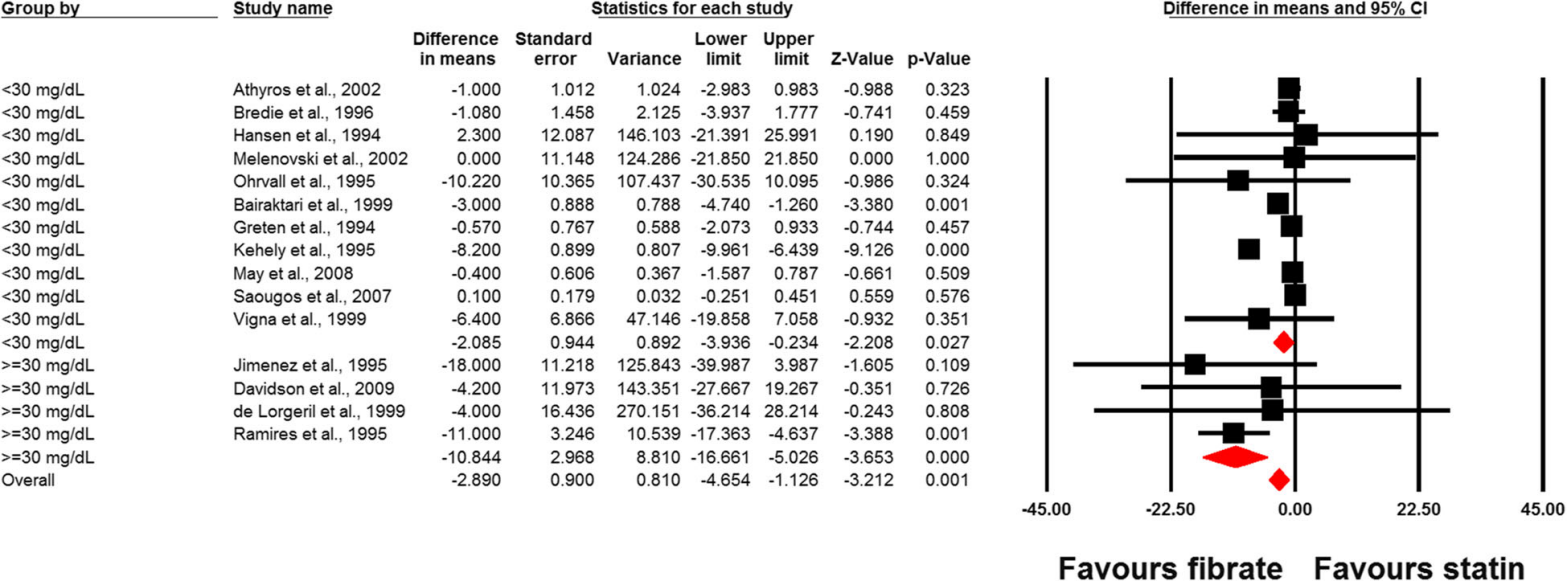
Forest plot detailing weighted mean difference and 95% confidence intervals for the impact of fibrate versus statin monotherapy in studies with baseline Lp(a) concentrations of < 30 mg/dL and ≥ 30 mg/dL

**Fig. 5:**
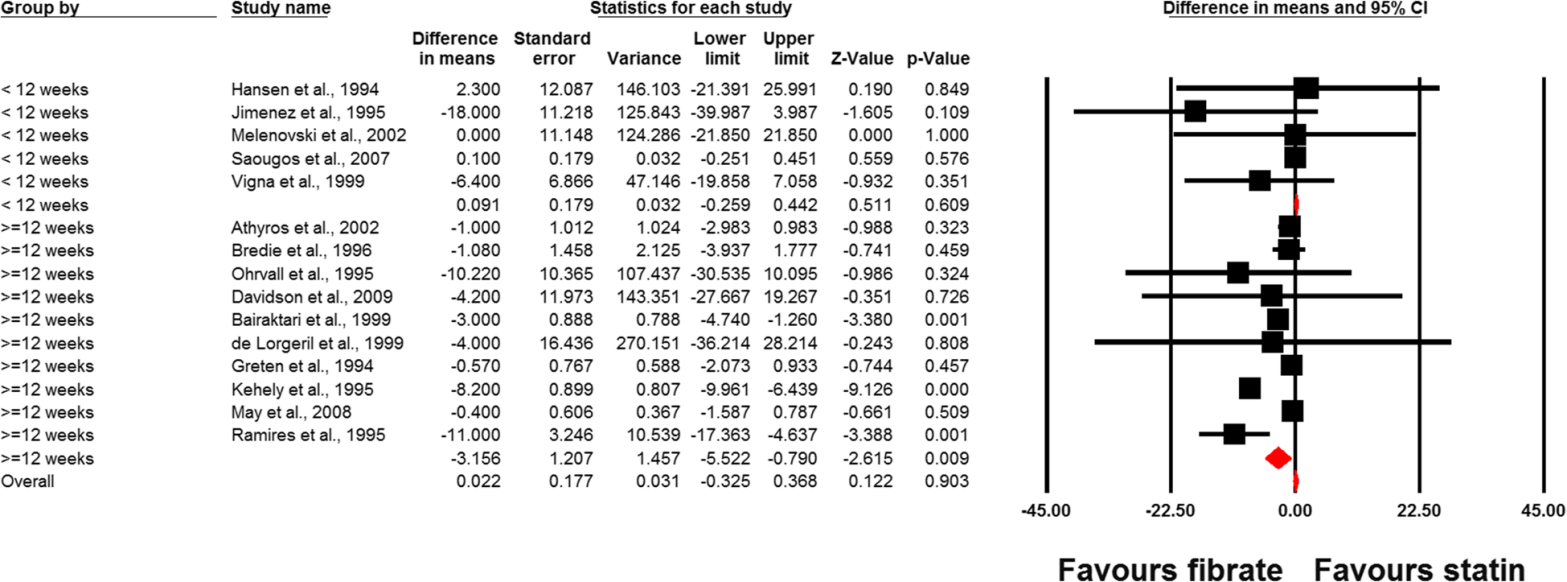
Forest plot detailing weighted mean difference and 95% confidence intervals for the impact of fibrate versus statin monotherapy on plasma Lp(a) concentrations in the subsets of trials < 12 and ≥ 12 weeks duration

In a single-arm analysis of randomized controlled study arms (without control group), statin therapy was found to increase plasma Lp(a) concentrations (WMD, 4.14 mg/dL; 95% CI, 0.15 to 8.12; *P* = 0.042), while the same effect was not observed with fibrates (WMD, 0.64 mg/dL; 95% CI, –1.59 to 2.87; *P* = 0.574). The lp(a)-raising effect of statins in single-arm analysis was diminished after exclusion of the trial with rosuvastatin (WMD, 4.56 mg/dL; 95% CI, –1.09 to 10.22; *P* = 0.113). Combination therapy with statins and fibrates did not exert a significant alteration in plasma Lp(a) concentrations (WMD, 4.52 mg/dL; 95% CI, –7.74 to 16.79; *P* = 0.470) (Fig. 2). Meta-analysis of data from 15 comparative trials showed a significantly greater effect of fibrates versus statins in reducing plasma Lp(a) concentrations (WMD, –2.70 mg/dL; 95% CI, –4.56 to –0.84; *P* = 0.004) (Fig. 3). This effect size was robust in sensitivity analysis and the overall estimated effect size was not significantly changed by the omission of a single study (Fig. 3). In the subgroup analysis, a greater effect of fibrates versus statins in reducing plasma Lp(a) levels was observed in the subset of trials with elevated baseline Lp(a) concentrations (≥30 mg/dL) (WMD, –10.84 mg/dL; 95% CI, –16.66 to –5.03; *P* < 0.001) compared with trials having baseline Lp(a) levels < 30 mg/dL (WMD, –2.08 mg/dL; 95% CI, –3.94 to –0.23; *P* = 0.027; *P* = 0.005 for between-subgroup comparison) (Fig. 4). With respect to treatment duration, the greater effect of fibrates versus statins in reducing plasma Lp(a) levels was observed in the subset of trials with ≥ 12 weeks length (WMD, –3.16 mg/dL; 95% CI, –5.52 to –0.79; *P* = 0.009); yet there was no significant difference between statins and fibrates in the subset of trials with a duration of < 12 weeks (WMD, +0.09 mg/dL; 95% CI, –0.26 to +0.44; *P* = 0.609; *P* = 0.008 for between-subgroup comparison) (Fig. 5).

#### Statin monotherapy versus statin/fibrate combination therapy

**Fig. 6:**
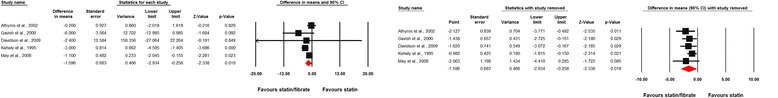
Forest plot detailing weighted mean difference and 95% confidence intervals for the impact of statin monotherapy versus statin/fibrate combination therapy on plasma Lp(a) concentrations. Right plot shows leave-one-out sensitivity analysis of the impact of statin monotherapy versus statin/fibrate combination therapy on plasma Lp(a) concentrations

Meta-analysis of data from five comparative trials showed a significantly greater effect of combination therapy with fibrates and statins versus statin monotherapy in reducing plasma Lp(a) concentrations (WMD, –1.60 mg/dL; 95% CI, –2.93 to –0.26; *P* = 0.019) (Fig. 6). In the sensitivity analysis, there was a partial sensitivity to the study by May et al. [57], which resulted in a borderline significant effect size (WMD, –2.06 mg/dL; 95% CI, –4.41 to +0.28; *P* = 0.085) (Fig. 6).

#### Fibrate monotherapy versus statin/fibrate combination therapy

**Fig. 7:**
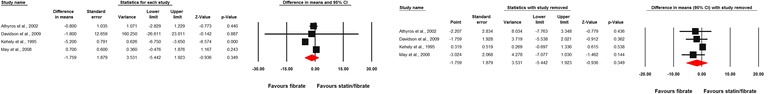
Forest plot detailing weighted mean difference and 95% confidence intervals for the impact of fibrate monotherapy versus statin/fibrate combination therapy on plasma Lp(a) concentrations. Right plot shows leave-one-out sensitivity analysis of the impact of fibrate monotherapy versus statin/fibrate combination therapy on plasma Lp(a) concentrations

Meta-analysis of data from four comparative trials did not suggest any significant difference between fibrate monotherapy and combination therapy with statins in terms of reducing plasma Lp(a) concentrations (WMD, –1.76 mg/dL; 95% CI, –5.44 to +1.92; *P* = 0.349) (Fig. 7). This effect size was robust in sensitivity analysis and the overall estimated effect size was not significantly changed by the omission of a single study (Fig. 7).

### Meta-regression

**Fig. 8:**
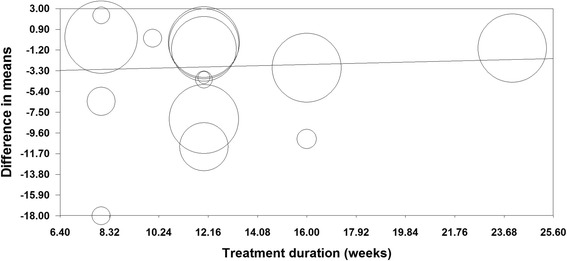
Meta-regression plots of the association between mean changes in plasma Lp(a) concentrations and duration of treatment. The size of each circle is inversely proportional to the variance of change

Meta-regression analysis was conducted to assess the association between changes in plasma Lp(a) concentrations with duration of treatment with statins and fibrates as a potential moderator. The impact of fibrates versus statins in reducing plasma Lp(a) concentrations was not found to be significantly associated with treatment duration (slope, +0.06; 95% CI, –0.40 to +0.53; *P* = 0.788) (Fig. 8).

### Publication bias

**Fig. 9:**
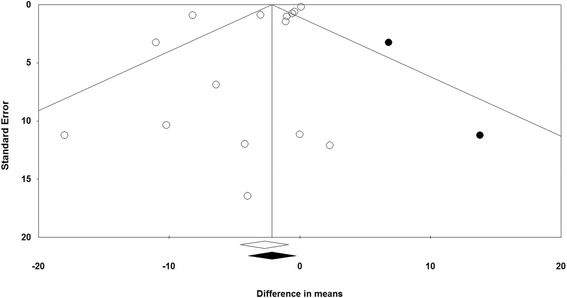
Funnel plot detailing publication bias in the studies reporting the impact of fibrate versus statin monotherapy on plasma Lp(a) concentrations. Open circles represent observed published studies; closed circles represent imputed unpublished studies

The funnel plot of the study precision (inverse standard error) by effect size (mean difference) was asymmetric and suggested potential publication bias. Although the results of Begg’s rank correlation (Kendall’s Tau with continuity correction = 0.02, *Z* = 0.10, two-tailed *P* value = 0.921) was not significant, Egger’s linear regression analysis suggested potential publication bias (intercept, –1.63; standard error, 0.76; 95% CI, –3.28 to +0.02; *t* = 3.01; df = 13.00; two-tailed *P* = 0.053). An attempt was made to address publication bias using trim-and-fill correction. Two potentially missing studies on the right side of funnel plot were imputed leading to a corrected effect size that was still significant (WMD, –2.12 mg/dL; 95% CI, –3.95 to –0.29). The “fail safe N” method indicated that 116 theoretically missing studies would be required to make the overall estimated effect size non-significant. Funnel plot of the impact of fibrates versus statins on plasma Lp(a) concentrations is illustrated in Fig. 9.

## Discussion

The findings of the present meta-analysis suggest that fibrates are more efficacious than statins in lowering plasma Lp(a) concentrations. In the absence of specific Lp(a)-lowering agents, statins and fibrates have been shown to reduce Lp(a) levels in hyperlipidemic subjects. However, the magnitude of the Lp(a)-lowering effect of these agents relative to each other has not been adequately investigated, and results of head-to-head comparative trials have not been fully clarified. Evidence of beneficial effect of statins on elevated plasma Lp(a) concentrations is still limited and variable [14, 66, 67]. In our single-arm analysis, statin therapy was found to increase plasma Lp(a) concentrations. This result is in contrast with some previous reports on the Lp(a)-lowering effect of statin therapy. While the limitation of our single-arm analysis in including only trials in which statins and fibrates were concomitantly studied should be considered, a possible reason for the observed increase in plasma Lp(a) concentrations could be attributed to the effect of rosuvastatin. There is evidence from previous trials indicating that, unlike atorvastatin and simvastatin, rosuvastatin therapy may significantly increase plasma Lp(a) levels [68, 69]. This is consistent with the results of our single-arm analysis, as excluding the only arm with rosuvastatin [65] from the analysis resulted in a non-significant overall effect of statin therapy on Lp(a) levels. Moreover, the results of the Justification for the Use of Statins in Prevention: An Intervention Trial Evaluating Rosuvastatin (JUPITER) trial showed a small but statistically significant positive shift in plasma Lp(a) levels following rosuvastatin therapy. The JUPITER trial also demonstrated that elevated plasma Lp(a) levels are a significant determinant for the residual cardiovascular risk in patients on optimal rosuvastatin therapy [70].

Statins have been shown to modestly decrease Lp(a) levels in individuals with familial hypercholesterolemia [71], but the mechanism of this effect remains elusive. This slight reduction could be explained by the strong genetic regulation of Lp(a) expression, as plasma Lp(a) concentration is significantly determined by genetic variability at the apo(a) gene locus or at other closely related loci [14]. With respect to fibrates, the effect on Lp(a) could be related to the induction of PPAR-α, and subsequent activation of farnesoid X receptor [72]. Inhibition of apoprotein(a) transcription by farsenoid X receptor has been shown to be mediated via translocation of the receptor to the nucleus, competitive inhibition of the binding of hepatocyte nuclear factor-4-α, and stimulation of fibroblast growth factor 19 expression in the intestine [73, 74]. Release of fatty acids from adipose tissue is another mechanism that may contribute to the Lp(a)-lowering effect of fibrates [55], but the specific mechanism remains unclear. Since Lp(a) may be bound to triglyceride-rich lipoproteins [75], the reduction of triglyceride-rich lipoproteins by fibrates could modify plasma Lp(a) concentrations [76], although this mechanism needs to be verified.

The results of two recent large randomized outcome trials with fenofibrate, Fenofibrate Intervention and Event Lowering in Diabetes (FIELD) [77] and Action to Control Cardiovascular Risk in Diabetes (ACCORD) [78], have not supported a benefit of fenofibrate on primary endpoints (myocardial infarction and death from coronary heart disease) in patients with type 2 diabetes taking statin. However, as our analysis revealed, the Lp(a)-lowering effect of fibrates might be more pronounced in individuals with elevated Lp(a) levels at baseline. FIELD and ACCORD trials were not designed to look at Lp(a) changes, and hyper-Lp(a) was not among the inclusion criteria of neither of these trials. Since subanalyses in subjects with atherogenic dyslipidemia in the above-mentioned trials have shown incremental benefits of adding fenofibrate to statin in diabetic patients, similar subanalyses in patients with hyper-Lp(a) could be worthwhile and deserve attention. However, it must be noted that the design in FIELD and ACCORD trials did not involve a head-to-head comparison of statins and fibrates, as opposed to the present meta-analysis.

Some limitations of the present analysis deserve acknowledgment. Several studies included in this meta-analysis did not provide sufficient information about the methods used for random sequence generation, allocation concealment, blinding of outcome assessment, and blinding of participants and personnel, resulting in uncertainty on their overall quality. Overall, there was a small population size in statin/fibrate combination therapy group, leading to a relatively low number of subjects in the pooled analysis; however, sensitivity analysis was conducted using the removal of one study (leave-one-out approach) in order to evaluate the influence of each study on the overall effect size. As another limitation, difference in the dose and duration of treatment as well as assays methods that were used to measure the Lp(a) concentrations might have introduced heterogeneity to the results. In this meta-analysis, the impact of this heterogeneity was tried to be minimized by applying a random-effects model and performing subgroup and meta-regression analyses. Finally, none of the included studies defined elevated Lp(a) concentrations among their inclusion criteria, which necessitates additional studies in patients with hyper-Lp(a).

## Conclusion

In conclusion, results of this meta-analysis suggest that fibrates have a significantly greater effect in reducing plasma Lp(a) concentrations compared with statins. Likewise, addition of fibrates to statins can enhance the Lp(a)-lowering effect of statins. Thus, combination therapy with fibrates and statins can provide an additional beneficial effect in decreasing the risk of developing cardiovascular disease by reducing apo(a) expression and enhancing Lp(a) clearance, especially in the subgroup of patients with hyper-Lp(a). Future investigations are recommended to explore the impact of other conventional Lp(a)-lowering therapies [79, 80] as well as novel lipid-modifying agents in comparison with fibrates and statins [81–84]. Moreover, further randomized head-to-head trials with different treatment durations could be helpful to clarify if prolongation of treatment could result in further reductions in plasma Lp(a) concentrations.

## References

[1] Djurovic S, Berg K (1997). Epidemiology of Lp(a) lipoprotein: its role in atherosclerotic/thrombotic disease. Clin Genet.

[2] Lippi G, Guidi G (2003). Lipoprotein(a): an emerging cardiovascular risk factor. Crit Rev Clin Lab Sci.

[3] Bennet A, Di Angelantonio E, Erqou S, Eiriksdottir G, Sigurdsson G, Woodward M, Rumley A, Lowe GD, Danesh J, Gudnason V (2008). Lipoprotein(a) levels and risk of future coronary heart disease: large-scale prospective data. Arch Intern Med.

[4] Kamstrup PR, Tybjærg-Hansen A, Steffensen R, Nordestgaard BG (2009). Genetically elevated lipoprotein(a) and increased risk of myocardial infarction. JAMA.

[5] Danik JS, Rifai N, Buring JE, Ridker PM (2006). Lipoprotein(a), measured with an assay independent of apolipoprotein(a) isoform size, and risk of future cardiovascular events among initially healthy women. JAMA.

[6] Scanu AM (2003). Lp(a) lipoprotein—coping with heterogeneity. N Engl J Med.

[7] Hajjar M, Katherine A, Nachman M, Ralph L (1996). The role of lipoprotein(a) in atherogenesis and thrombosis. Annu Rev Med.

[8] Rader DJ, Hoeg JM, Brewer HB (1994). Quantitation of plasma apolipoproteins in the primary and secondary prevention of coronary artery disease. Ann Intern Med.

[9] Kolski B, Tsimikas S (2012). Emerging therapeutic agents to lower lipoprotein(a) levels. Curr Opin Lipidol.

[10] Frank S, Hrzenjak A, Blaschitz A, Dohr G, Kostner G (2001). Role of various tissues in apo(a) fragmentation and excretion of fragments by the kidney. Eur J Clin Invest.

[11] Tsimikas S, Brilakis ES, Miller ER, McConnell JP, Lennon RJ, Kornman KS, Witztum JL, Berger PB (2005). Oxidized phospholipids, Lp(a) lipoprotein, and coronary artery disease. N Engl J Med.

[12] Collaboration ERF (2009). Lipoprotein(a) concentration and the risk of coronary heart disease, stroke, and nonvascular mortality. JAMA.

[13] Kamstrup PR (2010). Lipoprotein(a) and ischemic heart disease—a causal association? A review. Atherosclerosis.

[14] Lippi G, Targher G (2012). Optimal therapy for reduction of lipoprotein(a). J Clin Pharm Ther.

[15] Sahebkar A, Simental-Mendia LE, Stefanutti C, Pirro M (2016). Supplementation with coenzyme Q10 reduces plasma lipoprotein(a) concentrations but not other lipid indices: a systematic review and meta-analysis. Pharmacol Res..

[16] Serban MC, Sahebkar A, Mikhailidis DP, Toth PP, Jones SR, Muntner P, Blaha MJ, Andrica F, Martin SS, Borza C (2016). Impact of L-carnitine on plasma lipoprotein(a) concentrations: a systematic review and meta-analysis of randomized controlled trials. Sci Rep..

[17] Florentin M, Elisaf MS, Rizos CV, Nikolaou V, Bilianou E, Pitsavos C, Liberopoulos EN. l-Carnitine/Simvastatin Reduces Lipoprotein (a) Levels Compared with Simvastatin Monotherapy: A Randomized Double-Blind Placebo-Controlled Study. Lipids. 2017;52(1). doi:10.1007/s11745-016-4216-z. 10.1007/s11745-016-4216-z 27914033

[18] McKenney JM, Koren MJ, Kereiakes DJ, Hanotin C, Ferrand A-C, Stein EA (2012). Safety and efficacy of a monoclonal antibody to proprotein convertase subtilisin/kexin type 9 serine protease, SAR236553/REGN727, in patients with primary hypercholesterolemia receiving ongoing stable atorvastatin therapy. J Am Coll Cardiol.

[19] Desai NR, Kohli P, Giugliano RP, O’Donoghue ML, Somaratne R, Zhou J, Hoffman EB, Huang F, Rogers WJ, Wasserman SM (2013). AMG145, a monoclonal antibody against proprotein convertase subtilisin kexin type 9, significantly reduces lipoprotein(a) in hypercholesterolemic patients receiving statin therapy an analysis from the LDL-C assessment with proprotein convertase subtilisin kexin type 9 monoclonal antibody inhibition combined with statin therapy (LAPLACE)–thrombolysis in myocardial infarction (TIMI) 57 Trial. Circulation.

[20] Raal FJ, Giugliano RP, Sabatine MS, Koren MJ, Langslet G, Bays H, Blom D, Eriksson M, Dent R, Wasserman SM (2014). Reduction in lipoprotein(a) with PCSK9 monoclonal antibody evolocumab (AMG 145): a pooled analysis of more than 1,300 patients in 4 phase II trials. J Am Coll Cardiol.

[21] Stein EA, Giugliano RP, Koren MJ, Raal FJ, Roth EM, Weiss R, Sullivan D, Wasserman SM, Somaratne R, Kim JB (2014). Efficacy and safety of evolocumab (AMG 145), a fully human monoclonal antibody to PCSK9, in hyperlipidaemic patients on various background lipid therapies: pooled analysis of 1359 patients in four phase 2 trials. Eur Heart J.

[22] Zhang XL, Zhu QQ, Zhu L, Chen JZ, Chen QH, Li GN, Xie J, Kang LN, Xu B (2015). Safety and efficacy of anti-PCSK9 antibodies: a meta-analysis of 25 randomized, controlled trials. BMC Med..

[23] Navarese EP, Kolodziejczak M, Schulze V, Gurbel PA, Tantry U, Lin Y, Brockmeyer M, Kandzari DE, Kubica JM, D'Agostino RB (2015). Effects of proprotein convertase subtilisin/kexin type 9 antibodies in adults with hypercholesterolemia: a systematic review and meta-analysis. Ann Intern Med.

[24] Raal FJ, Giugliano RP, Sabatine MS, Koren MJ, Blom D, Seidah NG, Honarpour N, Lira A, Xue A, Chiruvolu P (2016). PCSK9 inhibition-mediated reduction in Lp(a) with evolocumab: an analysis of 10 clinical trials and the LDL receptor's role. J Lipid Res.

[25] Lippi G, Favaloro EJ (2011). Antisense therapy in the treatment of hypercholesterolemia. Eur J Intern Med.

[26] Panel NCEPNE (2002). Third Report of the National Cholesterol Education Program (NCEP) Expert Panel on Detection, Evaluation, and Treatment of High Blood Cholesterol in Adults (Adult Treatment Panel III) final report. Circulation.

[27] Grundy SM, Vega GL, Yuan Z, Battisti WP, Brady WE, Palmisano J (2005). Effectiveness and tolerability of simvastatin plus fenofibrate for combined hyperlipidemia (the SAFARI trial). Am J Cardiol.

[28] Cholesterol Treatment Trialists C, Mihaylova B, Emberson J, Blackwell L, Keech A, Simes J, Barnes EH, Voysey M, Gray A, Collins R (2012). The effects of lowering LDL cholesterol with statin therapy in people at low risk of vascular disease: meta-analysis of individual data from 27 randomised trials. Lancet.

[29] Rubins HB, Robins SJ, Collins D, Fye CL, Anderson JW, Elam MB, Faas FH, Linares E, Schaefer EJ, Schectman G (1999). Gemfibrozil for the secondary prevention of coronary heart disease in men with low levels of high-density lipoprotein cholesterol. Veterans Affairs High-Density Lipoprotein Cholesterol Intervention Trial Study Group. N Engl J Med.

[30] Adkins JC, Faulds D (1997). Micronised fenofibrate. Drugs.

[31] Heller F, Harvengt C (1983). Effects of clofibrate, bezafibrate, fenofibrate and probucol on plasma lipolytic enzymes in normolipaemic subjects. Eur J Clin Pharmacol.

[32] Martin G, Schoonjans K, Lefebvre A-M, Staels B, Auwerx J (1997). Coordinate regulation of the expression of the fatty acid transport protein and acyl-CoA synthetase genes by PPARα and PPARγ activators. J Biol Chem.

[33] Schoonjans K, Watanabe M, Suzuki H, Mahfoudi A, Krey G, Wahli W, Grimaldi P, Staels B, Yamamoto T, Auwerx J (1995). Induction of the acyl-coenzyme A synthetase gene by fibrates and fatty acids is mediated by a peroxisome proliferator response element in the C promoter. J Biol Chem.

[34] Caslake M, Packard C, Gaw A, Murray E, Griffin B, Vallance B, Shepherd J (1993). Fenofibrate and LDL metabolic heterogeneity in hypercholesterolemia. Arterioscler Thromb Vasc Biol.

[35] Mann CJ, Yen FT, Grant AM, Bihain BE (1991). Mechanism of plasma cholesteryl ester transfer in hypertriglyceridemia. J Clin Investig.

[36] Vu-Dac N, Schoonjans K, Kosykh V, Dallongeville J, Fruchart J-C, Staels B, Auwerx J (1995). Fibrates increase human apolipoprotein A-II expression through activation of the peroxisome proliferator-activated receptor. J Clin Investig.

[37] Alaupovic P, Heinonen T, Shurzinske L, Black DM (1997). Effect of a new HMG-CoA reductase inhibitor, atorvastatin, on lipids, apolipoproteins and lipoprotein particles in patients with elevated serum cholesterol and triglyceride levels. Atherosclerosis.

[38] Sehayek E, Butbul E, Avner R, Levkovitz H, Eisenberg S (1994). Enhanced cellular metabolism of very low density lipoprotein by simvastatin. A novel mechanism of action of HMG‐CoA reductase inhibitors. Eur J Clin Invest.

[39] Moher D, Liberati A, Tetzlaff J, Altman DG, Group P (2009). Preferred reporting items for systematic reviews and meta-analyses: the PRISMA statement. BMJ..

[40] Higgins JPT, Green S (2009). Handbook for Systematic Reviews of Interventions, Version 5.0.2 edn.

[41] Borenstein M, Hedges L, Higgins JPT (2005). Comprehensive Meta-analysis.

[42] Hozo SP, Djulbegovic B, Hozo I (2005). Estimating the mean and variance from the median, range, and the size of a sample. BMC Med Res Methodol..

[43] Sahebkar A (2013). Does PPAR gamma(2) gene Pro12Ala polymorphism affect nonalcoholic fatty liver disease risk? Evidence from a meta-analysis. DNA Cell Biol.

[44] Sahebkar A (2014). Are curcuminoids effective C-reactive protein-lowering agents in clinical practice? Evidence from a meta-analysis. Phytother Res.

[45] Ferretti G, Bacchetti T, Sahebkar A (2015). Effect of statin therapy on paraoxonase-1 status: a systematic review and meta-analysis of 25 clinical trials. Prog Lipid Res..

[46] Michalska-Kasiczak M, Sahebkar A, Mikhailidis DP, Rysz J, Muntner P, Toth PP, Jones SR, Rizzo M, Kees Hovingh G, Farnier M (2015). Analysis of vitamin D levels in patients with and without statin-associated myalgia – a systematic review and meta-analysis of 7 studies with 2420 patients. Int J Cardiol..

[47] Sahebkar A, Serban C, Mikhailidis DP, Undas A, Lip GYH, Muntner P, Bittner V, Ray KK, Watts GF, Hovingh GK, Rysz J, Kastelein, JJP, Banach M. Association between statin use and plasma d-dimer levels: A systematic review and meta-analysis of randomised controlled trials. Thromb Haemost. 2015;114(3):546–557. doi:10.1160/TH14-11-0937. 10.1160/TH14-11-0937 26017749

[48] Banach M, Serban C, Ursoniu S, Rysz J, Muntner P, Toth PP, Jones SR, Rizzo M, Glasser SP, Watts GF (2015). Statin therapy and plasma coenzyme Q10 concentrations--A systematic review and meta-analysis of placebo-controlled trials. Pharmacol Res..

[49] Duval S, Tweedie R (2000). Trim and fill: a simple funnel-plot-based method of testing and adjusting for publication bias in meta-analysis. Biometrics.

[50] Bairaktari ET, Tzallas CS, Tsimihodimos VK, Liberopoulos EN, Miltiadous GA, Elisaf MS (1999). Comparison of the efficacy of atorvastatin and micronized fenofibrate in the treatment of mixed hyperlipidemia. European J Cardiovasc Risk.

[51] Melenovsky V, Malik J, Wichterle D, Simek J, Pisarikova A, Skrha J, Poledne R, Stavek P, Ceska R (2002). Comparison of the effects of atorvastatin or fenofibrate on nonlipid biochemical risk factors and the LDL particle size in subjects with combined hyperlipidemia. Am Heart J.

[52] Athyros VG, Papageorgiou AA, Athyrou VV, Demitriadis DS, Kontopoulos AG (2002). Atorvastatin and micronized fenofibrate alone and in combination in type 2 diabetes with combined hyperlipidemia. Diabetes Care.

[53] Davidson MH, Rooney MW, Drucker J, Griffin HE, Oosman S, Beckert M, Investigators L-A (2009). Efficacy and tolerability of atorvastatin/fenofibrate fixed-dose combination tablet compared with atorvastatin and fenofibrate monotherapies in patients with dyslipidemia: a 12-week, multicenter, double-blind, randomized, parallel-group study. Clin Ther.

[54] Öhrvall M, Lithell H, Johansson J, Vessby B (1995). A comparison between the effects of gemfibrozil and simvastatin on insulin sensitivity in patients with non-insulin-dependent diabetes mellitus and hyperlipoproteinemia. Metabolism.

[55] Bredie S, Westerveld H, Knipscheer H, de Bruin T, Kastelein J, Stalenhoef A (1996). Effects of gemfibrozil or simvastatin on apolipoprotein-B-containing lipoproteins, apolipoprotein-CIII and lipoprotein (a) in familial combined hyperlipidaemia. Neth J Med.

[56] Kehely A, MacMahon M, Barbir M, Wray R, Hunt B, Prescott R, Thompson G (1995). Combined bezafibrate and simvastatin treatment for mixed hyperlipidaemia. QJM.

[57] May HT, Anderson JL, Pearson RR, Jensen JR, Horne BD, Lavasani F, Yannicelli HD, Muhlestein JB (2008). Comparison of effects of simvastatin alone versus fenofibrate alone versus simvastatin plus fenofibrate on lipoprotein subparticle profiles in diabetic patients with mixed dyslipidemia (from the Diabetes and Combined Lipid Therapy Regimen study). Am J Cardiol.

[58] Vigna G, Donega P, Passaro A, Zanca R, Cattin L, Fonda M, Pauciullo P, Marotta G, Fellin R, Gasparrini S (1999). Post-prandial effects of gemfibrozil vs simvastatin in hypercholesterolemic subjects with borderline hypertriglyceridemia. Nutr Metab Cardiovasc Dis.

[59] de Lorgeril M, Salen P, Bontemps L, Belichard P, Geyssant A, Itti R (1999). Effects of lipid-lowering drugs on left ventricular function and exercise tolerance in dyslipidemic coronary patients. J Cardiovasc Pharmacol.

[60] Hansen P, Meinertz H, Gerdes L, Klausen I, Faergeman O (1994). Treatment of patients with familial defective apolipoprotein B-100 with pravastatin and gemfibrozil: a two-period cross-over study. Clin Investig.

[61] Perez-Jimenez F, Hidalgo L, Zambrana JL, Arizon JM, Jimenez-Pereperez JA, Concha M, Espino A, Blanco J, Valles F, Lopez-Miranda J (1995). Comparison of lovastatin and bezafibrate on lipoprotein (a) plasma levels in cardiac transplant recipients. Am J Cardiol.

[62] Ramires JA, Mansur AP, Solimene MC, Maranhão R, Chamone D, da Luz P, Pileggi F (1995). Effect of gemfibrozil versus lovastatin on increased serum lipoprotein(a) levels of patients with hypercholesterolemia. Int J Cardiol.

[63] Ramires J, Spósito AC, Mansur AP, Solimene MC, Chamone D, da Luz PL, Pileggi F (1997). Gemfibrozil reduces elevated lipoprotein(a) levels in hypercholesterolemic patients. Arq Bras Cardiol.

[64] Greten H, Beil FU, Schneider J, Weisweiler P, Armstrong VW, Keller C, Klör H-U, von Hodenberg E, Weidinger G, Eskötter H (1994). Treatment of primary hypercholesterolemia: fluvastatin versus bezafibrate. Am J Med.

[65] Saougos VG, Tambaki AP, Kalogirou M, Kostapanos M, Gazi IF, Wolfert RL, Elisaf M, Tselepis AD (2007). Differential effect of hypolipidemic drugs on lipoprotein-associated phospholipase A2. Arterioscler Thromb Vasc Biol.

[66] Gonbert S, Malinsky S, Sposito AC, Laouenan H, Doucet C, Chapman MJ, Thillet J (2002). Atorvastatin lowers lipoprotein (a) but not apolipoprotein (a) fragment levels in hypercholesterolemic subjects at high cardiovascular risk. Atherosclerosis.

[67] Tziomalos K, Athyros VG, Wierzbicki AS, Mikhailidis DP (2009). Lipoprotein(a): where are we now?. Curr Opin Cardiol.

[68] Irudayam JB, Sivaraj R, Nirmala P (2014). Effect of statins on lipoprotein(a) in dyslipidemic patients. Int J Basic Clin Pharmacol.

[69] McKenney JM, Jones PH, Bays HE, Knopp RH, Kashyap ML, Ruoff GE, McGovern ME (2007). Comparative effects on lipid levels of combination therapy with a statin and extended-release niacin or ezetimibe versus a statin alone (the COMPELL study). Atherosclerosis.

[70] Khera AV, Everett BM, Caulfield MP, Hantash FM, Wohlgemuth J, Ridker PM, Mora S (2014). Lipoprotein(a) concentrations, rosuvastatin therapy, and residual vascular risk: an analysis from the JUPITER Trial (Justification for the Use of Statins in Prevention: an Intervention Trial Evaluating Rosuvastatin). Circulation.

[71] van Wissen S, Smilde TJ, Trip MD, de Boo T, Kastelein JJ, Stalenhoef AF (2003). Long term statin treatment reduces lipoprotein(a) concentrations in heterozygous familial hypercholesterolaemia. Heart.

[72] Staels B, Dallongeville J, Auwerx J, Schoonjans K, Leitersdorf E, Fruchart J-C (1998). Mechanism of action of fibrates on lipid and lipoprotein metabolism. Circulation.

[73] Chennamsetty I, Claudel T, Kostner KM, Baghdasaryan A, Kratky D, Levak-Frank S, Frank S, Gonzalez FJ, Trauner M, Kostner GM (2011). Farnesoid X receptor represses hepatic human APOA gene expression. J Clin Invest.

[74] Chennamsetty I, Claudel T, Kostner KM, Trauner M, Kostner GM (2012). FGF19 signaling cascade suppresses APOA gene expression. Arterioscler Thromb Vasc Biol.

[75] Cohn JS, Lam CW, Sullivan DR, Hensley WJ (1991). Plasma lipoprotein distribution of apolipoprotein(a) in the fed and fasted states. Atherosclerosis.

[76] Williams KJ, Fless GM, Petrie KA, Snyder ML, Brocia RW, Swenson TL (1992). Mechanisms by which lipoprotein lipase alters cellular metabolism of lipoprotein(a), low density lipoprotein, and nascent lipoproteins. Roles for low density lipoprotein receptors and heparan sulfate proteoglycans. J Biol Chem.

[77] Scott R, O'Brien R, Fulcher G, Pardy C, D'Emden M, Tse D, Taskinen MR, Ehnholm C, Keech A, Fenofibrate I (2009). Effects of fenofibrate treatment on cardiovascular disease risk in 9,795 individuals with type 2 diabetes and various components of the metabolic syndrome: the Fenofibrate Intervention and Event Lowering in Diabetes (FIELD) study. Diabetes Care.

[78] Ginsberg HN, Elam MB, Lovato LC, Crouse JR, Leiter LA, Linz P, Friedewald WT, Buse JB, Gerstein HC, ACCORD Study Group (2010). Effects of combination lipid therapy in type 2 diabetes mellitus. N Engl J Med.

[79] Jacobson TA (2013). Lipoprotein(a), cardiovascular disease, and contemporary management. Mayo Clin Proc.

[80] Kotani K, Sahebkar A, Serban C, Andrica F, Toth PP, Jones SR, Kostner K, Blaha MJ, Martin S, Rysz J (2015). Tibolone decreases lipoprotein(a) levels in postmenopausal women: a systematic review and meta-analysis of 12 studies with 1009 patients. Atherosclerosis.

[81] Sahebkar A, Chew GT, Watts GF (2014). New peroxisome proliferator-activated receptor agonists: potential treatments for atherogenic dyslipidemia and non-alcoholic fatty liver disease. Expert Opin Pharmacother.

[82] Sahebkar A (2013). Why it is necessary to translate curcumin into clinical practice for the prevention and treatment of metabolic syndrome?. Biofactors.

[83] Banach M, Aronow WS, Serban C, Sahabkar A, Rysz J, Voroneanu L, Covic A (2015). Lipids, blood pressure and kidney update 2014. Pharmacol Res..

[84] Sahebkar A, Serban MC, Gluba-Brzozka A, Mikhailidis DP, Cicero AF, Rysz J, Banach M (2016). Lipid-modifying effects of nutraceuticals: An evidence-based approach. Nutrition.

